# Supplementary material for: In vivo pharmacokinetic enhancement of monomeric Fc and monovalent bispecific designs through structural guidance
Source: Commun Biol. 2021 Sep 8;4:1048. doi: 10.1038/s42003-021-02565-5 (PMC8426389; doi:10.1038/s42003-021-02565-5)
Supplement: Supplementary file 2 — Supplementary Information [file 42003_2021_2565_MOESM2_ESM.pdf]

Supplementary Information

In vivo pharmacokinetic enhancement of monomeric Fc and monovalent bispecific designs through structural guidance

Lu Shan,[1#] Nydia Van Dyk,[1] Nantaporn Haskins,[1] Kimberly M. Cook,[2] Kim L. Rosenthal,[1] Ronit Mazor,[1*] Sonia Dragulin-Otto,[3] Yu Jiang,[4] Herren Wu,[1] William F. Dall'Acqua,[1] Martin J. Borrok,[1§] Melissa M. Damschroder,[1] Vaheh Oganesyan[1]

[1]Antibody Discovery and Protein Engineering, [2]Early Oncology, [3]Dosage Form Design and Development, and [4]Clinical Pharmacology and Safety Science, R&D, AstraZeneca, One MedImmune Way, Gaithersburg, MD 20878

[#]Current address: Denali Therapeutics, 161 Oyster Point Blvd, South San Francisco, CA 94080; [§]Current address: Janssen BioTherapeutics, Janssen Research and Development, 1400 McKean Road, Spring House, PA 19477; [*]Current address: Division of Cellular and Gene Therapies, FDA Center for Biologics Evaluation and Research 10903 New Hampshire Ave., Silver Spring, MD, USA.

Correspondence should be addressed to L.S. (email: shan@dnli.com) or to V.O. (email: vaheh.oganesyan@AstraZeneca.com).

Supplementary Methods

In Silico HLA binding prediction

Ten theoretical 23-25 mer peptides spanning 11 amino acids before and after each mutation in the monomeric Fc were analyzed. Five peptides represent WT sequences and five represent the mutations. In cases where two mutations were proximal, longer peptides spanning both mutations were analyzed. The binding affinity was predicted using the Immune Epitope Data Base MHC-II binding tool. The binding affinity of the peptides to 15 HLA-DR alleles, 6 HLA-DP alleles, and 6 HLA-DQ alleles were calculated using the IEDB-recommended method.[1] The binding affinity percentile rank of each overlapping 15-mer peptides comprising the 23-25mer peptides was calculated and the lowest value for each set of peptides was recorded. The lower the rank, the stronger the predicted binding. The binding threshold of 10% was imposed to score the number of alleles predicted to bind in high affinity. The peptide GQLLRDGATALELMVLNVPRLMTQD which is derived from Vatreptacog alfa, a mutant of factor VII that was found to have increased immunogenicity was used as a positive control.[2]

Cell binding assay and antibody-dependent cellular cytotoxicity (ADCC) assay

cMET-expressing cells were harvested and washed with PBS pH 7.2 and FACS buffer. Cells were incubated at 37°C with serial dilution of Onart-Fab-MFc1 and MetMab. Alexa Fluor 647-conjugated goat anti-human IgG affiniPure F(ab')2 fragment (Jackson ImmunoResearch, West Grove, Pennsylvania) at 1:400 dilution and DAPI (ThermoFisher) at 1:5000 were used to stain cells. BD FACSymphony™ Flow Cytometer (BD Biosciences, San Jose, CA) was used to measure cell binding.

The ADCC Reporter Bioassay kit (Promega, Madison, WI) was used according to the manufacture's manual. The high-expression cMET cell line (NCI H-1993) and low-expression cMET cell line (NCI H-358) were used as target cells; while the effector cells were part of the ADCC assay kit (Promega, Madison, WI). 10,000 effector cells and 30,000 target cells, with effector cells: target cells (E:T) ratios of 1:3, were added into each well in 96-well plates containing monovalent Onartuzumab fused to a monomeric Fc (Onart-Fab-MFc1) and one-arm

Onartuzumab Fab fused to knob-and-hole Fc (MetMab) with serial dilution concentrations accordingly.[3,4] The plates were incubated in a 37°C, 5% CO_2 incubator for 15 hours. After that, Bio-Glo kits (Promega# G7941) were used according to the manufacture's manual. Perkin Elmer Envision was used to measure the luminescence from each plate.

a

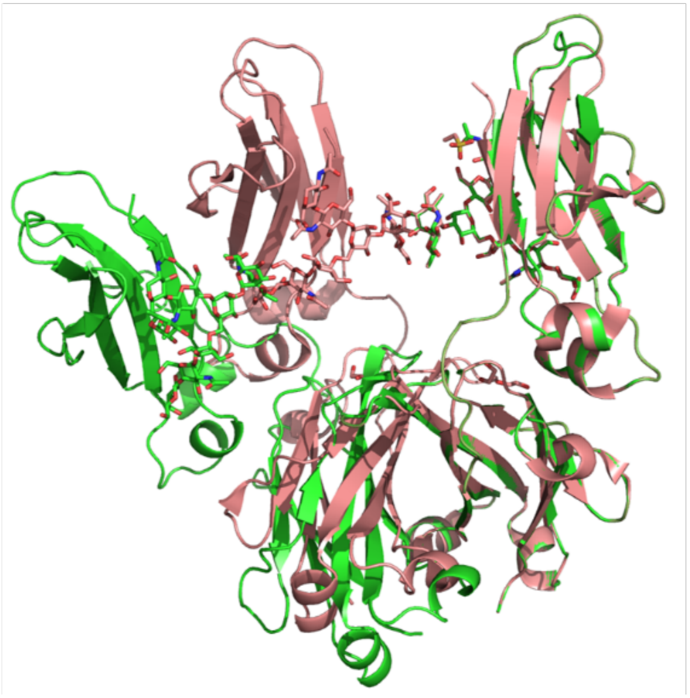

b

Hydrogen bonds between CH3 domains in IgG4 Fc.

##	CH3	Dist. [Å]	CH3
1	B:THR 366[OG1]	2.78	A:TYR 407[OH]
2	B:LYS 392[NZ]	2.97	A:SER 400[OG]
3	B:TYR 407[OH]	2.75	A:THR 366[OG1]
4	B:ARG 409[NH2]	2.37	A:ASP 399[OD2]
5	B:ASP 399[OD2]	2.86	A:ARG 409[NH1]
6	B:ASP 399[OD2]	2.82	A:ARG 409[NH2]

c

Hydrogen bonds between CH3 domains in T1 crystallographic dimer

##	CH3	Dist. [Å]	CH3*
1	A:THR 350[OG1]	2.91	A*:GLN 355[OE1]
2	A:THR 350[OG1]	2.98	A*:GLU 356[OE1]
3	A:THR 350[N]	3.05	A*:GLU 356[OE1]
4	A:LEU 440[N]	3.30	A*:GLU 356[OE2]
5	A:THR 350[OG1]	3.05	A*:GLU 356[OE2]
6	A:GLN 355[OE1]	2.91	A*:THR 350[OG1]
7	A:GLU 356[OE1]	2.98	A*:THR 350[OG1]
8	A:GLU 356[OE1]	3.05	A*:THR 350[N]
9	A:GLU 356[OE2]	3.05	A*:THR 350[OG1]
10	A:GLU 356[OE2]	3.30	A*:LEU 440[N]

Fig. S1 Structural comparison of the CH3 domain dimerization in IgG4 Fc and T1. **a** Superposition of IgG4 (PDB: 4c54; pink) and T1 (green) showed drastically different dimer formation. The CH3 interface area is 899 $Å^2$ in T1 and 1,051 $Å^2$ in IgG4 Fc. Also shown are the residues involved in hydrogen bond formation in **b** the IgG4 CH3 dimer interface versus **c** the T1 interface.

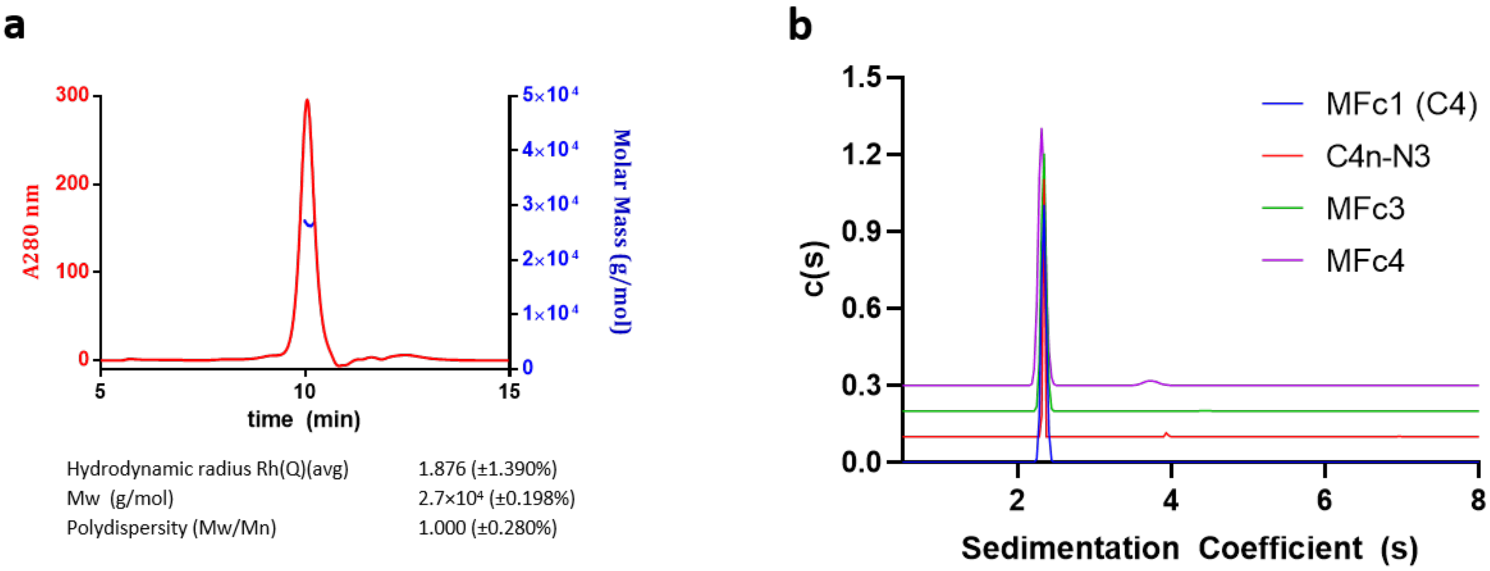

Fig. S2 a T1 was analyzed by size exclusion chromatography–multi-angle light scattering and showed good homogeneity and monomer state. To further investigate the oligomerization propensity of the T1 refinement variants at high concentrations, analytical ultracentrifugation was performed on these samples. **b** T1 was evaluated by analytical ultracentrifugation and showed almost exclusively monomer formation with a molecular weight of approximately 26–27 kDa, similar to MFc1, MFc3, and MFc4.

a

Position	ASA	BSA	ΔiG
LEU 251	26.19	20.08	-0.18
TYR 252	79	30.96	0.47
ILE 253	131.48	115.6	1.75
THR 254	133.04	80.8	0.58
LEU 309	109.07	22.77	0.36
HIS 310	37.76	24.51	-0.08
GLN 311	146.2	69.31	-0.23
LEU 314	36.13	2.01	0.03
HIS 433	145.81	9.59	-0.03
ASN 434	122.69	95.46	-0.05
HIS 435	73.09	45.67	-0.30
TYR 436	75.02	16.06	0.25
**TOTAL**	1115	533	2.67

b

Position	ASA	BSA	ΔiG
THR 250	4.22	1.47	-0.02
LEU 251	14.42	10.07	-0.08
TYR 252	77.44	35.01	0.55
ILE 253	134.74	130.06	1.63
THR 254	127.81	90.48	1.03
VAL 308	10.17	1.84	-0.02
LEU 309	126.4	29.46	0.47
HIS 310	23.39	18.69	0.16
GLN 311	106.17	20.01	-0.29
LEU 314	25.5	3.18	0.05
ASN 434	147.42	117.62	-0.38
HIS 435	48.2	42.33	-0.09
TYR 436	71.96	8.2	0.13
**TOTAL**	918	508	3.14

c

Position	ASA	BSA	ΔiG
THR 250	3.21	1.11	-0.01
LEU 251	14.41	10.23	-0.07
MET 252	73.85	26.7	0.43
ILE 253	144.49	139.14	1.73
SER 254	104.20	67.74	0.65
VAL 308	7.4	1.59	-0.02
LEU 309	127.92	28.62	0.46
HIS 310	26.22	23.57	0.14
GLN 311	103.72	14.35	-0.17
LEU 314	29.88	5.35	0.09
MET 428	19.64	3.85	0.06
TYR 434	197.41	133.89	1.22
HIS 435	47.55	41.35	-0.08
LEU 436	108.84	41.34	0.66
**TOTAL**	672	539	5.09

Fig. S3 Analysis of FcRn interactions with Fc variants **a** IgG1-Fc YTE, **b** MFc3, and **c** MFc4 on the PDBePISA web server.[5] Structural information from each of the FcRn complexes were loaded, and the differential solvation energy upon interface formation was calculated for each of the Fc variants. ASA, Accessible Surface Area, Å^2; BSA, Buried Surface Area, Å^2; ΔiG, differential solvation energy upon interface formation.

a

Peptides used in In Silico immunogenicity HLA binding analysis

Mutation	WT peptide	Mutant peptide
L351F	KGQPREPQVYTLPPSQEEMTKNQ	KGQPREPQVYTFPPSQEEMTKNQ
T366R	QEEMTKNQVSLTCLVKGFYPSDI	QEEMTKNQVSLRCLVKGFYPSDI
P395K	NGQPENNYKTTPPVLDSDGSFFL	NGQPENNYKTTKPVLDSDGSFRL
F405R_Y407E	TPPVLDSDGSFFLYSRLTVDKSRWQ	TKPVLDSDGSFRLESRLTVDKSRWQ
L351F_ S354E	QEEMTKNQVSLLPPSKGQPREPQVYT	QEEMTKNQVSLFPPEKGQPREPQVYT

Underlined amino acids represent the mutation in the amino acid sequence

b

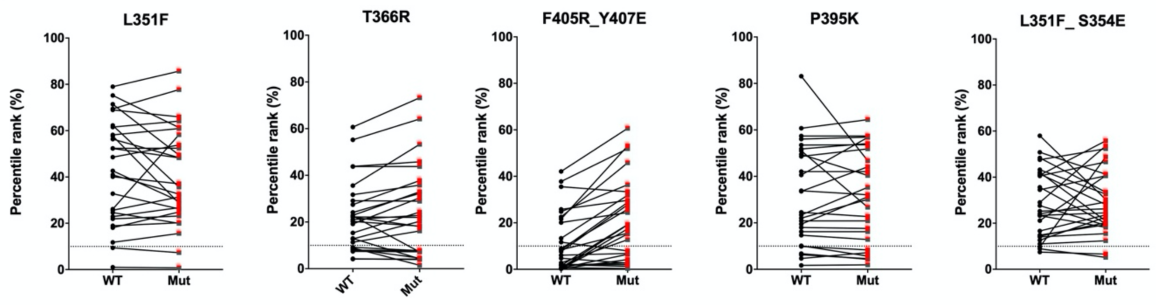

c

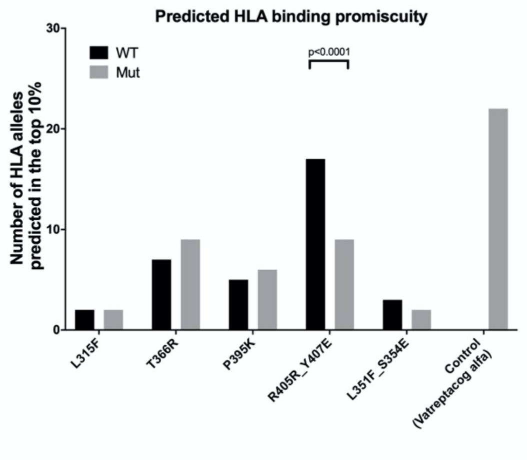

Fig. S4. ***In Silico* immunogenicity HLA binding analysis. a** Peptides used for *In silico* binding analysis. Theoretical 23 mer peptides containing 11 amino acids around each mutation in the monomeric Fc were analyzed. In cases where two mutations were proximal, longer peptides spanning both mutations were analyzed. The mutations are underscored. **b** Minimal predicted binding affinity between each peptide and 27 HLA class II alleles. For of each peptide, the binding affinity was predicted using the Immune Epitope Data Base MHC-II binding tool. The peptides were split into overlapping 15mer peptides, and the binding affinity was calculated to 15 HLA-DR alleles, 6 HLA-DP alleles, and 6 HLA-DQ alleles. The lowest binding affinity percentile rank of each pair of theoretical peptide and HLA allele was recorded: the lower the rank, the stronger the predicted binding and the higher the risk for T cell presentation and immunogenicity. **c** Promiscuous predicted binding. Immunogenicity is correlated with promiscuous HLA binding. The more promiscuous a peptide is, the higher the probability that it will be presented to a T cell and drive the immune response in a diverse population. The binding threshold of 10% was imposed to score the number of alleles predicted to bind in high affinity. The peptide GQLLRDGATALELMVLNVPRLMTQD which is derived from Vatreptacog alfa was used as a positive control.

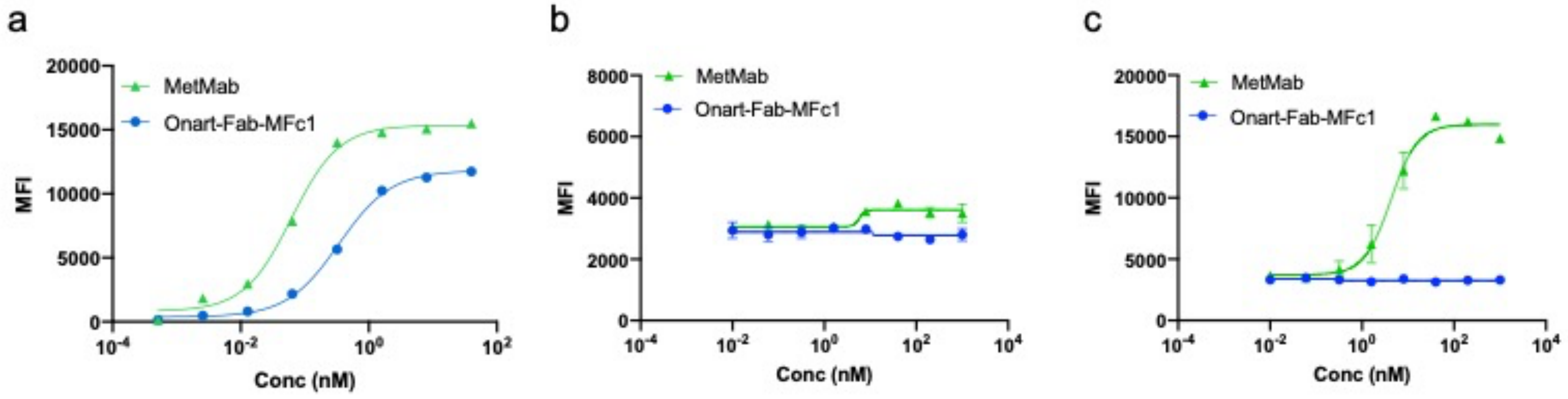

Fig. S5. **ADCC activity assay with monomeric and dimeric Fc fusion. a** Binding to cell-surface cMET was confirmed on NCI H-358 cells for Onart-Fab-MFc1 (blue), and MetMab (green). **b** Low cMET-expression NCI H-358 cells as target cells with Onart-Fab-MFc1 and MetMab. **c** High cMET-expression NCI H-1993 cells as target cells with Onart-Fab-MFc1 and MetMab.

References

1. Wang, P. et al. A systematic assessment of MHC class II peptide binding predictions and evaluation of a consensus approach. *PLoS Comput Biol* **4**, e1000048 (2008).
2. Lamberth, K. et al. Post hoc assessment of the immunogenicity of bioengineered factor VIIa demonstrates the use of preclinical tools. *Sci Transl Med* **9**(2017).
3. Merchant, M. et al. Monovalent antibody design and mechanism of action of onartuzumab, a MET antagonist with anti-tumor activity as a therapeutic agent. *Proc Natl Acad Sci U S A* **110**, E2987-96 (2013).
4. Shan, L. et al. Generation and Characterization of an IgG4 Monomeric Fc Platform. *PLoS One* **11**, e0160345 (2016).
5. Krissinel, E. & Henrick, K. Inference of macromolecular assemblies from crystalline state. *J Mol Biol* **372**, 774-97 (2007).
