# Supplementary material for: In vivo pharmacokinetic enhancement of monomeric Fc and monovalent bispecific designs through structural guidance
Source: Commun Biol. 2021 Sep 8;4:1048. doi: 10.1038/s42003-021-02565-5 (PMC8426389; doi:10.1038/s42003-021-02565-5)
Supplement: Supplementary file 3 — Description of Additional Supplementary Files [file 42003_2021_2565_MOESM3_ESM.pdf]

Description of Additional Supplementary Files

File name: Supplementary Data 1.

Description: Raw data file associated with Fig.6 of the manuscript.
